# DNMT3a-dermatopontin axis suppresses breast cancer malignancy via inactivating YAP

**DOI:** 10.1038/s41419-023-05657-8

**Published:** 2023-02-11

**Authors:** Danrong Ye, Yuying Wang, Xiaochong Deng, Xiqian Zhou, Diya Liu, Baian Zhou, Wenfang Zheng, Xuehui Wang, Lin Fang

**Affiliations:** 1 Department of Breast and Thyroid Surgery, Shanghai Tenth People’s Hospital, Tongji University School of Medicine, Shanghai, 200072 China; 2 Department of Breast Surgery, Guizhou Provincial People’s Hospital, Guiyang, 550002 China

**Keywords:** Breast cancer, Proteomics

## Abstract

Breast cancer (BC) is the most common malignant tumor in women worldwide, and its recurrence and metastasis negatively affect patient prognosis. However, the mechanisms underlying its tumorigenesis and progression remain unclear. Recently, the influence of dermatopontin (DPT), which is an extracellular matrix protein, has been proposed in the development of cancer. Here we found that DNMT3a-mediated DPT, promoter hypermethylation results in the downregulation of DPT expression in breast cancer and its low expression correlated with poor prognosis. Notably, DPT directly interacted with YAP to promote YAP Ser127 phosphorylation, and restricted the translocation of endogenous YAP from the cytoplasm to the nucleus, thereby suppressing malignant phenotypes in BC cells. In addition, Ectopic YAP overexpression reversed the inhibitory effects of DPT on BC growth and metastasis. Our study showed the critical role of DPT in regulating BC progression, making it easier to explore the clinical potential of modulating DPT/YAP activity in BC targeted therapies.

## Introduction

Breast cancer is considered the most common malignant tumor in women, and its incidence has increased in recent years [1]. In the United States, there will be approximately 290,560 cases of newly diagnosed BC in 2022, leading to a projected 43,780 deaths [2]. In China, the new estimated cases of BC alone account for 15% of all new cases in women, and breast cancer was the leading cause of cancer-related death among women younger than 45 years in 2015 [3]. Breast cancer is characterized by a high degree of heterogeneity and is clinically classified into four subtypes: Luminal A, Luminal B, HER2-positive, and triple-negative breast cancer [4]. Molecular studies on breast cancer diagnosis and treatment have been extensively carried out [5, 6]; however, the molecular mechanisms underlying breast cancer are still unknown. Thus, identifying potential therapeutic targets and biomarkers for diagnosis and prognosis should be a priority.

Dermatopontin (DPT) is a tyrosine-rich extracellular matrix (ECM) protein composed of 183 amino acid residues that was first extracted during the purification of dermatan sulfate proteoglycan from bovine skin [7]. Dermatopontin can accelerate collagen fibrillogenesis [8] and interact with decorin and TGF-β, thereby enhancing the biological activity of TGF-β [9, 10]. Studies have also indicated that DPT is involved in some diseases, such as myocardial infarction [11], obesity [12], nonalcoholic steatohepatitis [13], and several tumors [14–18]. Fu et al. discovered that DPT can inhibit the migration and invasion of hepatocellular carcinoma (HCC) cells by interacting with integrin proteins [19]. Furthermore, DPT is downregulated in papillary thyroid cancer (PTC) and it inhibits PTC proliferation through MEK-ERK-MYC signaling [17]. However, the role of DPT in BC progression remains unclear.

In the present study, low DPT expression was found in BC and was relevant to a poor prognosis in BC patients. We discovered that DNMT3a-mediated DNA methylation of the DPT promoter region was responsible for DPT downregulation. Decreased DPT levels resulted in BC cell growth, migration, and invasion by interacting with YAP and inactivating the Hippo/YAP pathway. These findings offer a novel approach for breast cancer diagnosis and treatment.

## Results

### DPT is downregulated in BCs and predicts a poor prognosis

**Fig. 1:**
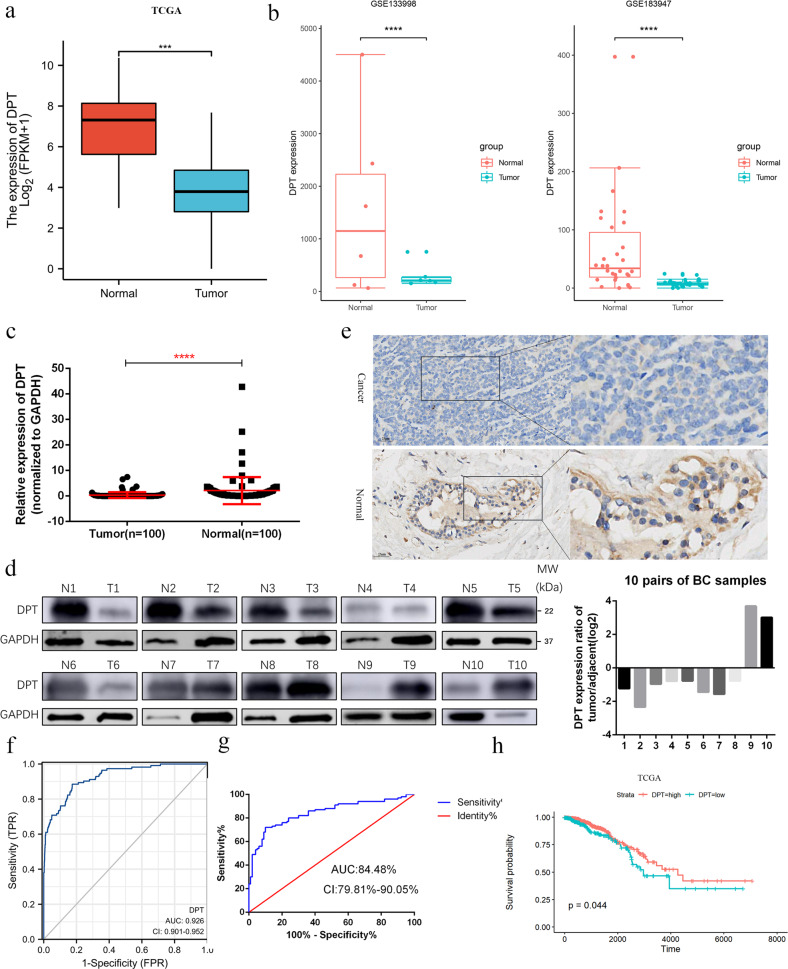
Low expression of DPT in BCs predicts a poor prognosis. **a** Expression levels of DPT in BC and normal breast tissues from The Cancer Genome Atlas (TCGA) database. **b** Expression of DPT in the GSE133998 and GSE183947 BC cohorts. **c** Quantitative RT-PCR analysis of DPT mRNA expression in 100 paired BC tissues and non-cancerous tissues. **d** Representative western blotting analysis showed DPT expression in ten pairs of human BC tissues and adjacent nontumorous tissues and their grayscale analysis were conducted. **e** Representative IHC staining image of DPT in paired BC tissues and non-cancerous tissues. **f** ROC curve for DPT expression to diagnose BC in the TCGA cohort. **g** ROC curve for DPT expression to diagnose BC in 100 breast cancer patients. **h** OS analysis of DPT expression in BC patients. ***P < 0.001, ****P < 0.0001. All experiments were repeated three times.

To explore the expression of DPT in BC, we searched The Cancer Genome Atlas (TCGA) breast carcinoma database for differential expression between tumor tissues and normal mammary gland tissues. DPT expression was significantly lower in BC tissues than in normal breast tissues (Fig. 1a). Subsequent gene expression analysis of DPT from the GEO dataset (GSE133998 and GSE183947) confirmed this result (Fig. 1b). We then analyzed the mRNA and protein levels of DPT in paired BC tissues and matched non-cancerous breast tissues by qRT-PCR and western blotting, and found that DPT was downregulated in most BC (8 out of 10 matched tumor/normal tissues analyzed) (Fig. 1c, d). Immunohistochemistry (IHC) staining also confirmed that DPT was downregulated in BC (Fig. 1e). Moreover, DPT was detected by qRT-PCR in BC and normal breast epithelial cells (MCF-10A), and decreased DPT expression were discovered in BC cells (Additional file: Fig. S1a). Furthermore, we plotted a receiver operator characteristic (ROC) curve to assess the diagnostic value of DPT expression in BCs. It’s AUC was 92.6% in the TCGA cohort (CI 90.1–95.2%) (Fig. 1f) while it was 84.48% in the validated cohort (CI 79.81–90.05%) (Fig. 1g). We then analyzed the association between DPT expression and clinicopathological features of patients with BC. The patients were divided into low and high DPT expression groups based on the median value. As shown in the TCGA cohort, age at diagnosis (>60 years *P* = 0.026), basal-like tumor subtype (*P* < 0.001), and tumor size (*P* = 0.023) were significantly associated with lower DPT expression (Table 1). And in the validated cohort, increased DPT expression was negatively correlated with tumor size (*P* = 0.039) (Table 2). Survival analysis indicated that BC patients with low DPT expression exhibited worse overall survival (OS) (Fig. 1h). Taken together, these results suggest that DPT is significantly downregulated in BC and may be associated with BC progression.

**Table 1:** The relationship between DPT expression and clinicopathological characteristics in TCGA cohort.

Characteristics	Expression of DPT, number (%)		
	Low expression (*n* = 538) (%)	High expression (*n* = 539) (%)	*P*-value
*Age at diagnosis, y*			
Mean ± SD	59.19 ± 13.50	57.64 ± 12.87	0.055
≤60 y	279 (51.86)	316 (58.63)	0.026*
>60 y	259 (48.14)	223 (41.37)	
*Subtype*			<0.001*
Luminal A	199(36.99)	298 (55.29)	
Luminal B	137(25.46)	59 (10.95)	
Basal-like	111(20.63)	60 (11.13)	
HER-2+	34(6.32)	44 (8.16)	
*Tumor size in cm*			
≤2 cm	121 (22.49)	154 (28.57)	0.023*
>2 cm	415 (77.14)	384 (71.24)	
*Lymph node metastasis*			0.122
≤3 metastatic sites	440 (81.63)	423 (78.48)	
>3 metastatic sites	87 (16.17)	107 (19.85)	
*Metastasis*			0.585
M0	458 (85.13)	438 (81.26)	
M1	12 (2.23)	9 (1.67)	
*AJCC disease stage*			
I + II	402 (74.72)	391 (72.54)	0.405
III + IV	127 (23.61)	139 (25.79)	

*P-value < 0.05.

**Table 2:** The relationship between DPT expression and clinicopathological characteristics in validated cohort.

Characteristics	Expression of DPT, number (%)		
	Low expression (*n* = 50) (%)	High expression (*n* = 50) (%)	*P*-value
*Age at diagnosis, y*			
Mean ± SD	61.94 ± 11.83	58.78 ± 10.65	0.164
≤60 y	22 (44.00)	29 (58.00)	0.161
>60 y	28 (56.00)	21 (42.00)	
*Tumor size in cm*			
≤2 cm	14 (28.00)	24 (48.00)	0.039*
>2 cm	36 (72.00)	26 (52.00)	
*Lymph node metastasis*			0.779
≤3 metastatic sites	43 (81.63)	42 (78.48)	
>3 metastatic sites	7 (16.17)	8 (19.85)	
*Metastasis*			0.646
M0	48 (96.00)	47 (94.00)	
M1	2 (4.00)	3 (6.00)	
*AJCC disease stage*			
I + II	40 (80.00)	39 (78.00)	0.806
III + IV	10 (20.00)	11 (22.00)	
*Estrogen receptor status*			0.509
Positive	37 (74.00)	34 (68.00)	
Negative	13 (26.00)	16 (32.00)	
*Progesterone receptor status*			1.000
Positive	28 (56.00)	28 (56.00)	
Negative	22 (44.00)	22 (44.00)	
*HER2 status*			0.790
Positive	8 (16.00)	9 (18.00)	
Negative	42 (84.00)	41 (82.00)	

*P-value < 0.05

### DPT is regulated by DNA methylation

**Fig. 2:**
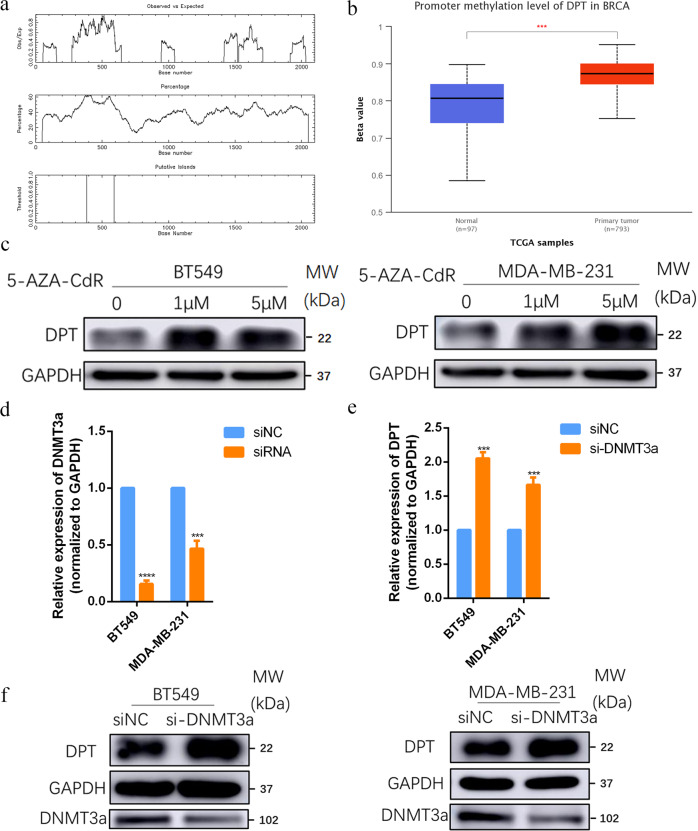
DNMT3a-mediated hypermethylation of DPT lead to its downregulation in breast cancer. **a** Prediction of CpG islands in the DPT’s promoter region by methylation software analysis. The upper panel showed Observed/Expected ratio of CpG dinucleotide >0.60; The middle panel showed Percent C + Percent G > 50.00; The bottom panel showed the location of putative CpG island. **b** Methylation level of the promoter region of DPT from The Cancer Genome Atlas (TCGA) database (The Beta value indicates level of DNA methylation). **c** BT549 and MDA-MB-231 **c**ells were treated with 5-AZA-CdR at indicated concentrations for 72 h, and DPT expression was examined by western blotting. **d** Inhibition of DNMT3a expression by siRNA in BT549 and MDA-MB-231 cells was verified by qRT-PCR. **e**, **f** DPT expression was analyzed by qRT-PCR and western blotting after transfection of DNMT3a siRNA in BT549 and MDA-MB-231 cells. ***P < 0.001, ****P < 0.0001.

To clarify the mechanism responsible for the downregulation of DPT in BC, we predicted CpG islands in the promoter region of DPT using methylation software analysis [20] (Fig. 2a). By analyzing TCGA database in UALCAN (http://ualcan.path.uab.edu/), we found that the methylation level of the DPT promoter region was significantly higher in BC tissues than in adjacent normal breast tissues (Fig. 2b). To determine whether DPT downregulation caused by promoter hypermethylation, BT549, and MDA-MB-231 cells were treated with the demethylation agent 5-AZA-CdR. We found that 5-AZA-CdR significantly enhanced the DPT mRNA and protein levels in BC cells (Fig. 2c and Fig. S1b). Furthermore, an analysis of the TCGA datasets revealed that DPT negatively correlated with DNMT3a at the RNA level (Fig. S1c). Knockdown of DNMT3a by specific small interfering RNA (siRNA) (Fig. 2d) restored the expression of DPT at the mRNA and protein level (Fig. 2e, f), indicating that DNMT3a could mediate DPT promoter methylation in BC. The chromatin immunoprecipitation (ChIP)-qPCR assay was conducted to further confirm the DNMT3a’ direct hypermethylation regulation on DPT promoter. As shown in Fig. S1d, DNMT3a bond to the DPT promoter region. In conclusion, the above data demonstrated that DPT is modulated by DNA-methylation in BC.

### Restored DPT suppresses BC cell growth, migration, and invasion

**Fig. 3:**
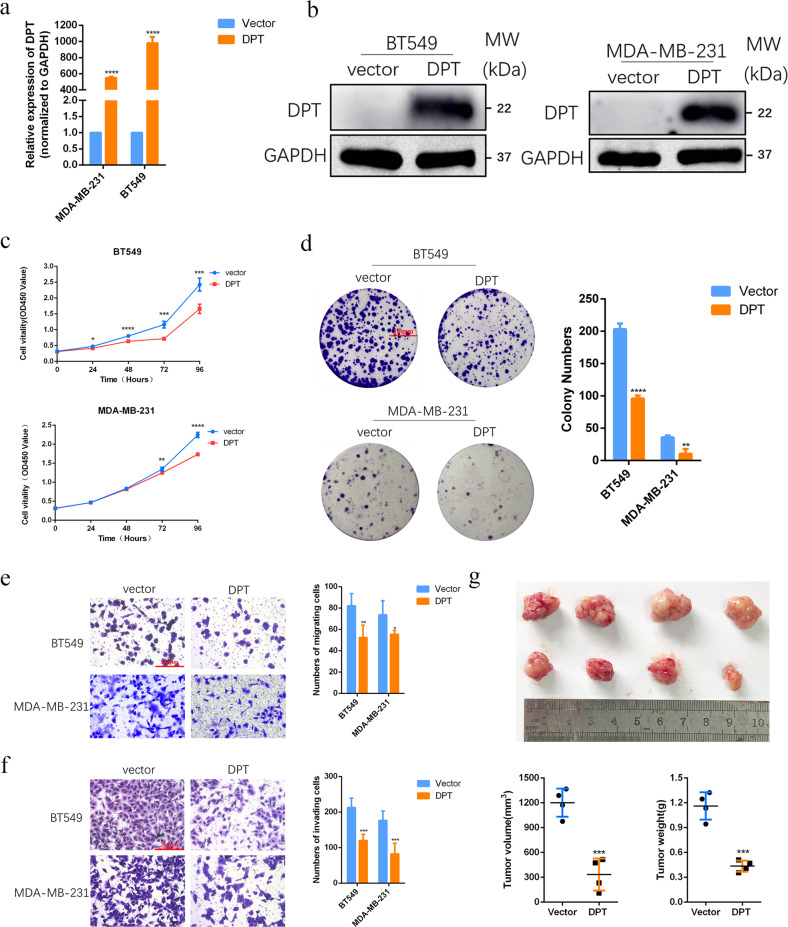
Restoring expression of DPT suppresses BC cell growth, migration, and invasion in vitro and tumor growth in vivo. **a**, **b** The mRNA and protein levels of DPT in BT549 and MDA-MB-231 cells with DPT overexpression were measured by qRT-PCR and western blotting. **c** CCK-8 assays were performed to compare the proliferative capacity of BT549 and MDA-MB-231 cells after overexpression of DPT. **d** Colony formation assays were conducted to compare the colony-forming abilities of BT549 and MDA-MB-231 cells after overexpression of DPT. **e** Transwell migration assays were conducted to compare the migratory abilities of BT549 and MDA-MB-231 cells after overexpression of DPT. **f** Transwell invasion assays were conducted to compare the invasive abilities of BT549 and MDA-MB-231 cells after overexpression of DPT. **g** Up panel, representative image of the xenograft tumors dissected from vector and DPT groups at the endpoint of the experiments (day 30). Down panel, the tumor volume and weight were measured. *P < 0.05, **P < 0.01, ***P < 0.001, ****P < 0.0001.

To identify the biological function of DPT in BC cells, we constructed BC cell lines that stably overexpress DPT. Firstly, DPT expression in stably transfected cells was confirmed by qRT-PCR and western blotting (Fig. 3a, b). A series of in vitro and in vivo experiments were performed to verify the role of DPT in BC cells. The CCK8 assay showed that DPT overexpression significantly inhibited cell proliferation in both the BT549 and MDA-MB-231 cell lines (Fig. 3c). Colony formation experiments were used to study the long-term effects of DPT on BC cell proliferation. Consistently, fewer colonies were observed in the DPT overexpression group than in the vector group (Fig. 3d). Furthermore, we evaluated the effects of DPT on BC cell migration and invasion. We observed that DPT overexpression dramatically inhibited the migration and invasion abilities of BT549 and MDA-MB-231 cells relative to those of the control (Fig. 3e, f). The above results of the in vitro assays indicated that DPT acts as a tumor suppressor in BC cell lines. To further determine the function of DPT in anti-tumorigenesis in vivo, MDA-MB-231 cells stably overexpressing DPT or transfected with vector cells were subcutaneously injected into nude mice. As expected, tumor growth was remarkably suppressed in the DPT-overexpressing group compared with the vector group. In addition, tumor volumes and weights were significantly reduced in the DPT-overexpressing groups compared with the control groups at the end of observation (Fig. 3g). In brief, these in vitro and in vivo data showed that DPT serves as a vital anti-oncogene by inhibiting tumorigenesis in breast cancer.

### DPT downregulation enhances BC cell growth, migration, and invasion

**Fig. 4:**
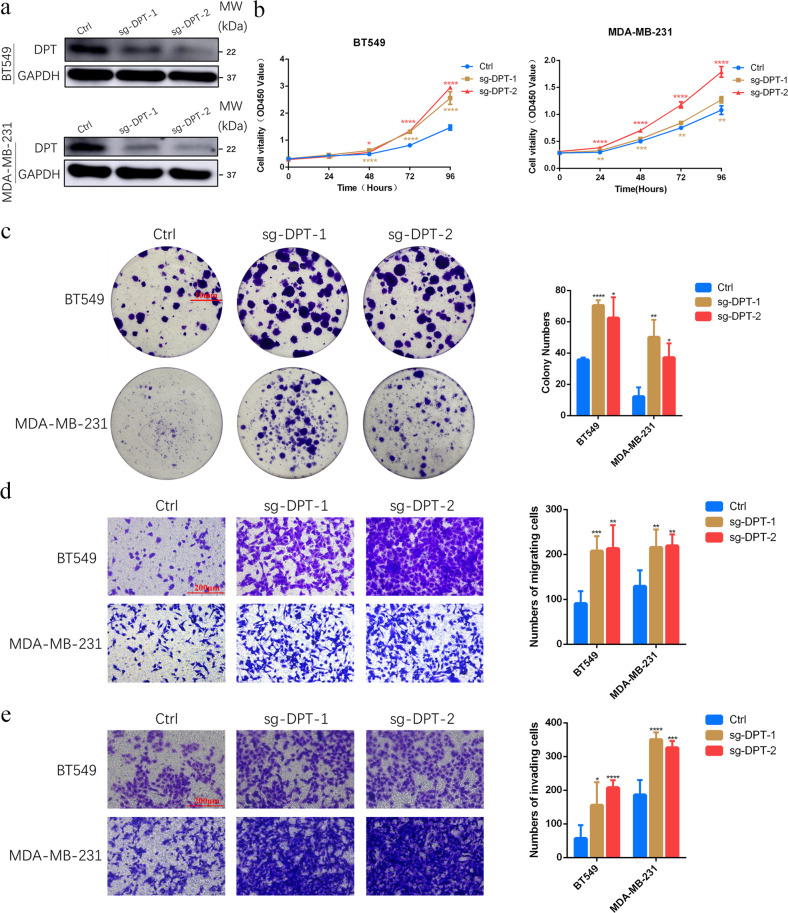
Abolishing expression of DPT promotes BC cell growth, migration, and invasion. **a** The protein levels of DPT in BT549 and MDA-MB-231 cells with DPT knockout were measured by western blotting. **b** CCK-8 assays were performed to detect the proliferative capacity of BT549 and MDA-MB-231 cells with DPT knockout. **c** Colony formation assays were conducted to determine the colony-forming abilities of BT549 and MDA-MB-231 cells with DPT knockout. **d** Transwell migration assays were conducted to detect the migratory abilities of BT549 and MDA-MB-231 cells with DPT knockout. **e** Transwell invasion assays were conducted to detect the invasive abilities of BT549 and MDA-MB-231 cells with DPT knockout. *P < 0.05, **P < 0.01, ***P < 0.001, ****P < 0.0001.

To further explore the functional role of DPT in BC, we used CRISPR-Cas9 to knock out DPT in BT549 and MDA-MB-231 cells. Knockout efficiency was confirmed using western blotting (Fig. 4a). Knockout of DPT markedly enhanced proliferation and colony formation ability of BC cells (Fig. 4b, c). The potential for cell migration and invasion was also evaluated. Compared with the control cell groups, DPT knockout dramatically facilitated migration and invasion in BT549 and MDA-MB-231 cells (Fig. 4d, e). Collectively, these data demonstrated that DPT functions as a key factor in antitumor activity.

### DPT interacts with YAP and inhibits the activation of Hippo/YAP pathway in BC cells

**Fig. 5:**
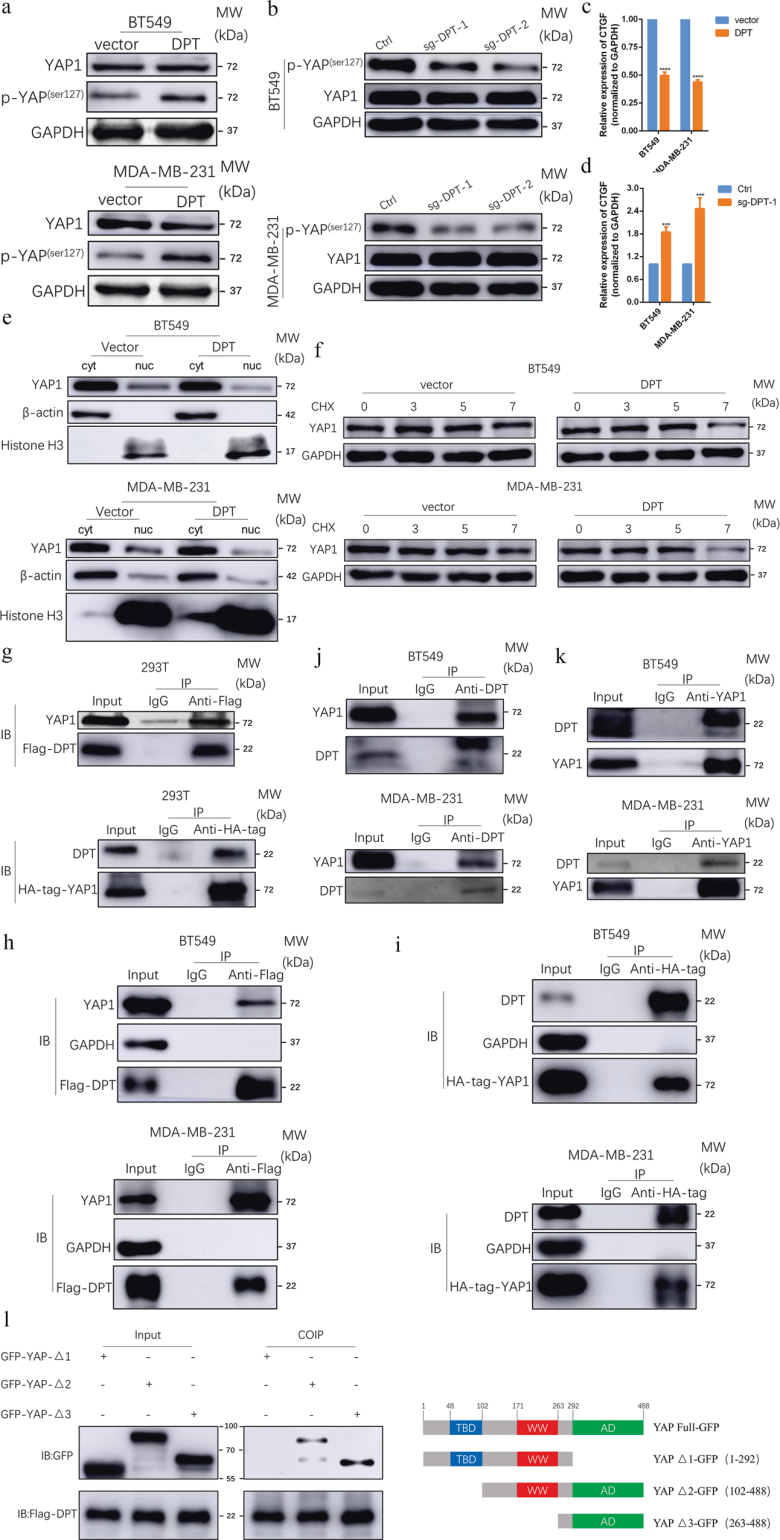
DPT stably interacts with YAP and suppresses the Hippo/YAP signaling pathway in BC cells. **a** Western blotting showed expression level of total YAP and p-YAP in vector and DPT-OE cells. **b** total YAP and phosphorylated YAP (Ser127) were evaluated by western blot in ctrl and DPT-KO cells. **c** The mRNA levels of CTGF in DPT overexpression and vector cells. **d** The mRNA levels of CTGF in ctrl and DPT-KO cells. **e** Nuclear-cytoplasm separation assay indicated that DPT overexpression regulated YAP translocation between nuclear and cytoplasm. **f** Protein stability assay indicated the expression of YAP in different time points after Cycloheximide (CHX, 100 μg/ml) treatment in DPT overexpression and vector cells. **g**–**i** Immunoprecipitation showed the interaction between exogenous DPT and YAP proteins. **j**, **k** Immunoprecipitation assay showed the interaction between endogenous DPT and YAP proteins in BT549 and MDA-MB-231 cells. **l** Western blot showed that GFP-tagged YAP were precipitated by in vitro transcribed DPT in 293T cells. Right panel: graphic illustration of the YAP domain structure. ***P < 0.001, ****P < 0.0001.

To further explore the possible mechanism underlying DPT-mediated suppression of BC cell growth, migration and invasion, we performed pathway enrichment analysis of the genes co-expressed with DPT. The results showed that the co-expressed genes were significantly enriched in Hippo signaling (*P* < 0.01 Fig. S1e), implying that hippo signaling may be involved in DPT-mediated BC suppression. Yes-associated protein (YAP) is a key downstream member of the Hippo pathway and is phosphorylated at the residue serine127 by MST1/2 and LATS1/2, retaining it in the cytoplasm, then accelerating its ubiquitination degradation and inactivating transcription. Therefore, we examined YAP activity after DPT overexpression or knockout. As expected, DPT overexpression increased the phosphorylation level of YAP (Fig. 5a), whereas DPT knockout distinctly decreased the phosphorylation of YAP in BC cells (Fig. 5b), when compared to the control. Furthermore, the mRNA levels of a typical YAP target gene, *CTGF*, were detected by qRT-PCR. Exogenous DPT impeded the expression of CTGF at the mRNA level, whereas knockout of DPT activated its expression (Fig. 5c, d). In addition, according to the results of the cytoplasmic/nuclear protein extraction assay, we found that DPT overexpression impaired the nuclear accumulation of YAP in BT549 and MDA-MB-231 cells (Fig. 5e), and increased nuclear expression was observed in DPT knockout cells (Fig. S1f). A cycloheximide chase experiment showed that DPT overexpression could shorten the half-life of YAP (Fig. 5f), and DPT overexpression increased ubiquitination of YAP (Fig. S1g). To further determine the molecular mechanism and binding proteins of DPT, a coimmunoprecipitation (CoIP) assay was performed. The CoIP of exogenous proteins indicated that DPT directly interacted with YAP (Fig. 5g–i). Consistently, CoIP/western blotting was designed to further ascertain the interaction between endogenous YAP and DPT in BT549 and MDA-MB-231 cells using an Anti-YAP1 or Anti-DPT antibodies (Fig. 5j, k). In addition, double immunofluorescent staining using confocal microscopy showed co-localization of DPT and YAP in cytoplasm of BT549 and MDA-MB-231 cells (Fig. S1h). To identify potential DPT-interacting YAP domains, several YAP domain-deletion mutants were constructed. Coimmunoprecipitation experiments with multiple mutants of YAP demonstrated that the 292-488 amino acid region of YAP was responsible for binding with DPT (Fig. 5l). Taken together, these data suggest that DPT physically interacts with YAP and inhibits its activation.

### DPT suppresses malignant phenotypes of breast cancer in a YAP-dependent manner

**Fig. 6:**
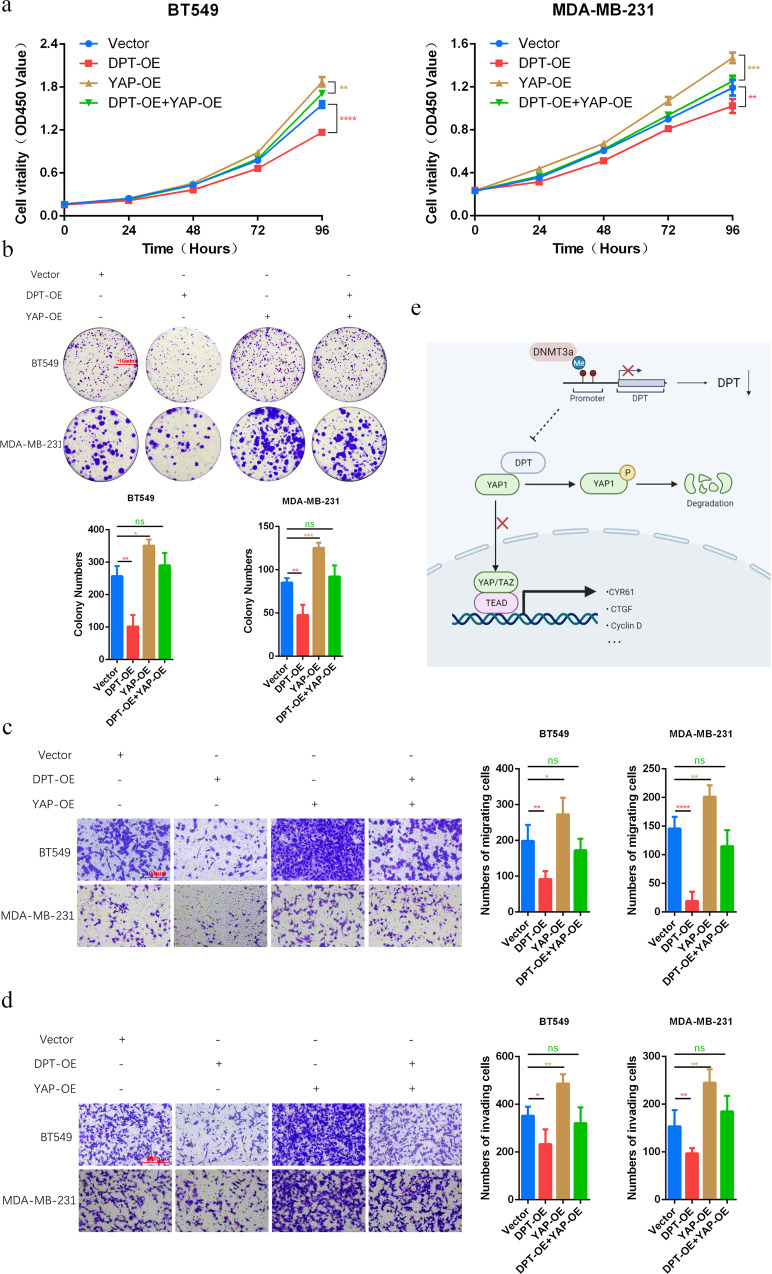
DPT-mediated tumor suppression in BC cells is dependent on YAP. **a** CCK-8 assays were conducted to evaluate the effects of DPT and YAP co-transfection on the proliferation of MDA-MB-231 and BT549 cells. **b** Colony formation assays were performed in either DPT-OE or YAP1-OE BT549 and MDA-MB-231 cells. Column diagrams (lower panel) showed colony numbers of each group. **c** Left panel: transwell migration assays to evaluate the effects of YAP overexpression on DPT-overexpressed BC cells were shown; right panel: statistical analysis of left panel. **d** Left panel: transwell invasion assays conducted in DPT/YAP-rescued cells were shown; right panel: statistical analysis of left panel. **e** A schematic model of the proposed molecular mechanisms show that DNMT3a/DPT/YAP signaling inhibits tumor malignancy in BC. DNMT3a-mediated promoter hypermethylation lead to DPT downregulation. DPT directly interacts with YAP and thus suppresses YAP translocating from cytoplasm to the nucleus. Finally, it inhibits downstream transcriptional activation and impedes tumor development. *P < 0.05, **P < 0.01, ***P < 0.001, ****P < 0.0001.

We then performed rescue experiments to further confirm if YAP functions in DPT-mediated inhibition of breast cancer progression. Ectopic DPT was transfected into BC cells with or without YAP overexpression. CCK8 assays and colony formation experiments showed that YAP overexpression eliminated DPT-mediated suppression of cell proliferation (Fig. 6a, b). In addition, YAP reversed DPT-induced suppression of cell migration and invasion (Fig. 6c, d). Taken together, these results demonstrate that YAP inhibition is required for DPT-induced tumor suppression in breast cancer cells.

## Discussion

Dermatopontin, an important component of the extracellular matrix (ECM), is probably responsible for matrix assembly and cell-matrix interactions. It has been reported to have functions in the development and progression of tumors. Yamatoji et al. found that DPT was downregulated in human oral squamous cell carcinoma (OSCC) and was relevant to cellular adhesion and invasiveness [14]. A recent study illustrated that DPT repressed HCC proliferation by inactivating the Wnt signaling pathway [18]. Here, we showed that the expression of DPT was drastically downregulated in BC tissues whether in the TCGA cohort or in the validated cohort consisting of 100 paired BC tissues, and negatively correlated with tumor subtype and tumor size. In addition, ROC curve analysis showed a high diagnostic value for DPT, and patients with lower DPT expression exhibited poor survival. These data strongly indicate that DPT can serve as a novel diagnostic and prognostic marker for BC. Furthermore, gain-of-function of DPT obviously suppressed the malignant biological properties of BC, such as cell growth, migration, and invasion, while knockout of DPT markedly increased cancer cell proliferation and metastasis. It’s well established that obesity is a risk factor for different cancers and the increasing prevalence of obesity in women, especially in young and middle-aged women, may lead to higher incidence of cancers such as postmenopausal breast cancer [21]. Obesity-associated dysfunctional adipose tissue (AT) also secretes pro-oncogenic factors such as TNFα, IL-6, IL-1β and provides a carcinogenesis-promoting microenvironment for breast cancer development and progression; it is therefore a fundamental factor of postmenopausal breast cancer development [22]. Recently, the influence of DPT in AT remodeling and inflammation has been proposed [23]. A great variety of biological functions in both, physiological and pathological processes have been attributed to DPT due to its interaction with the transforming growth factor-β (TGF-β) and decorin together with its binding to integrin α3β1 and syndecan [9, 10, 24]. Understanding the cellular and molecular basis of the interaction will allow DPT to be targeted specifically within the breast cancer. Further studies in larger cohorts to improve our insight of the role of DPT in obesity-associated breast cancer are needed and more studies on the dissection of signaling mechanisms of DPT-mediated angiogenesis might unravel novel targets for BC.

Epigenetic modifications play an increasingly important role in the initiation and progression of malignant tumors. DNA methylation of gene promoter regions inactivates or silences gene expressions [25, 26]. Emerging researches have shown that aberrant DNA methylation of CpG islands is relevant to loss of gene expression, development, chemotherapy sensitivity, and cancer stem cell (CSC) epigenetic regulation of BC [27–31]. Hence, identifying novel anti-oncogenes induced by promoter methylation may be an alternative approach for diagnostic and therapeutic evaluation. Aberrant DPT hypermethylation of CpG islands in the promoter region has been discovered in HCC [19]. Similarly, we illustrated that DPT promoter is hypermethylated in BC and that DPT expression is restored after treatment with the demethylation agent 5-AZA-CdR. The methylation of CpG islands in the promoter is catalyzed by DNA methyltransferases (DNMTs), including DNMT1, DNMT3a, and DNMT3b [32]. Through informative analysis TCGA data, we found that DNMT3a expression negatively correlated with DPT expression in BC. In addition, DNMT3a knockdown restored DPT expression and DNMT3a bond to the DPT promoter region, indicating that the downregulation of DPT expression is due to DNMT3a-mediated aberrant hypermethylation of its promoter. Recently, epigenetic-based therapies including drugs that act on specific enzymes, such as DNMTs, have been studied [33, 34]. Therefore, increasing DPT expression levels using demethylating agents may have therapeutic value for BC.

The Hippo/YAP pathway is a highly conserved tumor suppressor signaling pathway in mammals and plays an important role in organ size regulation, carcinogenesis, tissue regeneration, and stem cell function. Dysregulation of the Hippo/YAP pathway has been reported in many cancers, including BC, lung carcinoma, colorectal cancer, ovarian cancer, hepatocellular carcinoma, and prostate cancers [35–38], and is often associated with patients’ poor survival [39–41]. The core components of the Hippo pathway are Mst1/2, Sav1, Lats1/2, and Mob1, which phosphorylate YAP, the major downstream effector of the Hippo/YAP pathway. Phosphorylated YAP accumulates in the cytoplasm by binding to 14‑3‑3 and accelerates its ubiquitination degradation, thereby inhibiting the growth-promoting and anti-apoptotic functions of YAP. The Hippo pathway negatively regulates YAP activity via cascade of phosphorylation reactions. In contrast, when the Hippo pathway is inhibited, YAP is transported to the nucleus and binds to transcription factors such as TEADs, to promote the expression of target genes. In this study, pathway enrichment analysis based on the co-expression gene network revealed that the enriched pathway included the Hippo signaling pathway. We found that DPT directly bound to YAP and particularly bound to the 292-488 amino acid region of YAP to promote its cytoplasmic retention. In addition, we demonstrated that the interaction between DPT and YAP leads to YAP phosphorylation at Ser127 in BC cells, which reduces the nuclear translocation of YAP, accelerates ubiquitination degradation and inactivates its transcriptional function. Rescue assays further indicated that YAP overexpression could reverse the inhibitory effect of DPT on the growth, migration, and invasion of BC cells.

In conclusion, our study revealed that DPT is under-expressed in BC, which results from DNMT3a-mediated aberrant hypermethylation of its promoter, and functions as a tumor suppressor. DPT directly binds to YAP, thereby promoting its phosphorylation, restricting its nuclear translocation, and promoting its degradation (Fig. 6e). These findings also underline the diagnostic and prognostic value of DPT in breast cancer and may provide a promising strategy for BC treatment.

## Materials and methods

### Patients and samples

A total of 100 BC tissues and matched non-cancerous tissues were collected from breast cancer patients undergoing surgery in Shanghai Tenth People’s Hospital between 2019 and 2022. None of the patients received chemotherapy, radiotherapy, or other neoadjuvant therapy preoperatively. The clinicopathological and molecular characteristics of the patients were described in detail in Table 2. The study received approval from the Ethics Committee of Shanghai Tenth People’s Hospital and was conducted in accordance with Declaration of Helsinki. Informed consent was obtained from all patients before enrollment. All tissue samples were stored in liquid nitrogen as soon as they were resected.

### Cell culture

Human breast cancer cell lines (BT549, MDA-MB-231, HCC1937, SK-BR-3, and MCF-7), human breast normal epithelial cell line (MCF10A) and human embryonic kidney cell line (HEK293T) were obtained from the Chinese Academy of Sciences (Shanghai, China). BT549 and HCC1937 were cultured in RPMI 1640 with 10% Fetal Bovine Serum (FBS, Gibco) and 1% penicillin-streptomycin mixture (Servicebio). MDA-MB-231, SK-BR-3, MCF-7, and 293T were maintained in DMEM medium with high glucose plus 10% Fetal Bovine Serum (FBS, Gibco) with 1% penicillin-streptomycin mixture (Servicebio). MCF10A cells were cultured in a special medium for MCF10A (Procell). All cell lines described above were cultivated at 37 °C with 5% CO_2_.

### Cell transfection

The DPT and YAP overexpression lentivirus and negative control were purchased from Generay Biotechnology (Shanghai, China). Three mutant plasmid of YAP were constructed by IBSBio (Shanghai, China). The transfection of plasmids was performed using Lipofectamine 3000 (Invitrogen), according to the manufacturer’s instructions. To obtain the DPT stable overexpressing cell line, BT549 and MDA-MB-231 cells were infected with LV-DPT and LV-NC, and selected using 2 μg/ml puromycin.

### Quantitative real-time polymerase chain reaction (qRT-PCR)

TRIzol reagent (Invitrogen) was applied to extract total RNA of BC tissues and the cultured cells according to the manufacturer’s instructions. Then RNAs were reverse transcribed into cDNA by HiScript® II Q RT SuperMix for qPCR (+gDNA wiper) (Vazyme, Nanjing, China). qRT-PCR was performed using Hieff UNICON®qPCR SYBR Green MasterMix (Yeasen, China) in an QuantStudio™ Dx Real-Time PCR Instrument (Applied Biosystems, USA). The results were normalized to GAPDH. The primers for qRT-PCR were as follow: DPT (forward GGGGCCAGTATGGCGATTATG and reverse CGGTTCAAATTCACCCACCC); DNMT3a (forward ACGATTGCTAGACTGGGATAATG and reverse AGTAAGCAGGCCAGGTAGA); GAPDH (forward GGAGCGAGATCCCTCCAAAAT and reverse GGCTGTTGTCATACTTCTCATGG).

### Immunohistochemistry (IHC)

Breast cancer tissues and matched non-cancerous breast tissues samples were fixed with 4% paraformaldehyde and embedded in paraffin. Then they were cut into 4 μm sections and stained following the routine protocol. The primary antibodies were the following: anti-DPT (1:50, 10537-1-AP, Proteintech, USA).

### CCK8 and colony formation assay

The ability of cell proliferation was evaluated by CCK8 assay. Transfected BC cells (BT549 1.5 × 10^3^ cells/well; MDA-MB-231 2.0 × 10^3^ cells/well) were seeded into 96-well plates. CCK8 reagent (10 μl) (Yeasen, China) was added into each well and cells were incubated for 2 h at 37 °C in 5% CO_2_. Then absorbance at 450 nm was recorded. For colony formation assay, transfected cells (BT549 0.8 × 10^3^ cells/well; MDA-MB-231 1.0 × 10^3^ cells/well) were seeded into 6-well plates. The medium was replaced every 3–4 days. After culturing 7 to 14 days, colonies were fixed in 4% paraformaldehyde and stained with 0.01% crystal violet.

### Cell migration and invasion assay

For migration assay, the treated cells (BT549 4 × 10^4^ cells; MDA-MB-231 5 × 10^4^ cells) were seeded into the upper chamber of Transwell® permeable supports (Corning Costar Corp, Cambridge, USA), whereas, for invasion assay, BC cells (BT549 8 × 10^4^ cells; MDA-MB-231 1.0 × 10^5^ cells) were placed in the upper chamber with matrigel (BD Biosciences, USA). After incubation for 19 h, non-migrated or invaded cells were gently wiped off with a cotton swab and cells in the bottom chamber were fixed with 4% paraformaldehyde, and stained with 0.01% crystal violet. Then five visions were photographed by a microscope at a magnification of ×200 and counted with ImageJ software. The experiments were replicated in triple.

### 5-Aza-2’-deoxycytidine (5-AZA-CdR, MCE, USA) treatment

BT549 and MDA-MB-231 cells were seeded into 6-well plates and exposed to 0, 1, and 5 μM of 5-AZA-CdR for 72 h. The 5-AZA-CdR was replenished every day.

### CRISPR-Cas9 system

Two pairs of sgRNA sequences targeting for the exon of DPT were designed and then inserted into the pCAG-T7-cas9+gDNA-pgk-puro-T2A-GFP vector. Then plasmids of sgDPT-1/sgDPT-2 and negative control plasmid (Ctrl) were transfected into BC cells using Lipofectamine 3000 (Invitrogen) according to the manufacturer’s instructions. After 48 h, cells were filtered by 2 μg/ml puromycin for 1–2 weeks. The sequences of sgRNAs were as follow: sg-DPT-1 (forward CCGGATAATCGCCATACTGGCCCC and reverse AACGGGGCCAGTATGGCGATTATC) and sg-DPT-2 (forward CCGGATCATGACTACAGCGATGAT and reverse AACATCATCGCTGTAGTCATGATC).

### Protein extraction and western blot analysis

The treated cells were lysed using RIPA buffer containing protease and phosphatase inhibitors. Equal amounts of protein samples were separated on SDS-PAGE and transferred onto nitrocellulose membranes (Pall, Port Washington, USA). Next, the membranes were blocked with 5% nonfat milk for 1 h at room temperature. Then the membranes were incubated with primary antibodies at 4 °C overnight and incubated with secondary antibody for 1 h at room temperature. The information of antibodies were as follow: DPT (ab255823, Abcam, UK), Yes-associated protein 1 (YAP1, A1002, ABclonal, China), Phospho-YAP (Ser127) (#13008, CST, USA), GAPDH (#5174, CST, USA), β-actin (AC026, ABclonal, China), Histone H3 (A2348, ABclonal, China), HA tag (ab236632, Abcam, UK), DYKDDDDK Tag (#14793, CST, USA), and GFP-Tag (AE011, ABclonal, China).

### Coimmunoprecipitation (CoIP) assay

The immunoprecipitation lysis buffer with PMSF (abs955, Absin, China) was employed to lyse cells. Total protein lysates (500 μl) were incubated with primary antibody or immunoglobulin G (IgG). Then the mixtures were rotated at 4 °C overnight and 5 μl of protein A and 5 μl of protein G were added, followed by an overnight incubation at 4 °C. The pellets after centrifugation were washed three times with wash buffer. A 1× SDS sampling buffer was added to the samples, which were heated at 100 °C for 5 min and then analyzed by western blot.

### Xenograft subcutaneous implantation

Four-week-old BALB/c nu/nu female mice were purchased from Shanghai Lingchang Biotechnology (Shanghai, China). A total of 1 × 10^7^ MDA-MB-231 cells after stable transfected with DPT or vector were subcutaneously injected into each mouse. The mice were euthanized on the 30th days after injection. Then the tumor weight and volume were measured. All animal studies were approved by the Institutional Animal Care and Use Committee of the Shanghai Tenth People’s hospital.

### Statistical analysis

All data analyses involved in this article were performed via the SPSS software (version 22.0; SPSS) and the GraphPad Prism (6.0). The differences in expression between BC tissues and normal breast tissues were evaluated by Mann–Whitney U test. The relationships between DPT expression and patents’ clinicopathologic characteristics were analyzed via Chi-square test or Fisher’s exact test. Survival curves were compared using the log-rank test. Student’s t-test was used for analyzing normally distributed data, which were expressed as mean ± SD. Values of *P* < 0.05 were considered statistically significant.

## Supplementary information

Supplementary Information

Supplementary Figure 1

reproducibility checklist

## Data Availability

Correspondence and requests for materials should be addressed to LF.
