# Supplementary material for: DNMT3a-dermatopontin axis suppresses breast cancer malignancy via inactivating YAP
Source: Cell Death Dis. 2023 Feb 11;14(2):106. doi: 10.1038/s41419-023-05657-8 (PMC9922281; doi:10.1038/s41419-023-05657-8)
Supplement: Supplementary file 1 — Supplementary Information [file 41419_2023_5657_MOESM1_ESM.docx]

Supplementary Information for:

**DNMT3a-mediated DPT suppresses breast cancer malignancy via inactivating YAP**

**Supplemental Materials and Methods**

**Chromatin immunoprecipitation**

ChIP was performed using BersinBio^TM^ Chromatin Immunoprecipitation (ChIP) Kit (BersinBio, Catalog Bes5001) according to the manufacturer’s protocol. Briefly, 2×10^7^ cells were harvested for chromatin preparation. The cells were fixed with 1% formaldehyde for 10 min at room temperature, washed twice with PBS, collected and resuspended in lysis buffer. The DNA was fragmented by sonication, and Anti-DNMT3a (sc-365769, santa cruz, USA) or IgG antibodies were added into lysate to generate the protein/DNA complexes. Finally, the complexes were reverse cross-linked to free immunoprecipitated DNA. ChIP-qPCR was performed using the QuantStudio™ Dx Real-Time PCR Instrument with SYBR green dye. The primers were as follow: forward TTTAGTAGAGGCGGGGTTTCAC and reverse GTGGTGGCTCATGCTTGTAATC.

**Immunofluorescence (IF)**

Cells, which stably overexpressed DPT, were plated onto 14mm glass cover slides. Then cells were fixed with 4% paraformaldehyde for 20 minutes, and permeabilized with 0.5% Triton X-100. After blocking with 5% Bovine Serum Albumin (BSA), cells were incubated with Anti-YAP1 (YAP1, A1002, ABclonal, China) and Anti-FLAG (#8146, CST, USA) for overnight at 4℃, and then Goat Anti-Rabbit IgG H&L (Alexa Fluor® 488) (ab150077, Abcam, UK) and Goat Anti-Mouse IgG H&L (Cy3®) (ab97035, Abcam, UK) at room temperature for 1 hour. The Nuclei were stained with DAPI. Representative images were observed using confocal microscopy (Carl Zeiss Jena, Germany).

**Supplementary figure legend**

**Fig S1 a** Quantitative RT-PCR analysis of DPT mRNA expression in normal breast epithelial cell line MCF10A and breast cancer cell lines MDA-MB-231, BT549, HCC1937, SK-BR-3 and MCF-7. **b** BT549 and MDA-MB-231 cells were treated with 5-AZA-CdR at indicated concentrations for 72 h, and DPT expression was examined by qRT-PCR. **c** TCGA data analysis showed the inverse correlation of DPT and DNMT3a based on RNA expression. **d** The ChIP assays showed that DNMT3a bond to the DPT promoter region. **e** Pathway enrichment analysis on the co-expressed genes of DPT. **f** Nuclear-cytoplasm separation assay indicated that DPT knockout regulated YAP translocation between nuclear and cytoplasm. **g** DPT overexpression increased ubiquitination of YAP. **h** Immunofluorescence showed co-localization of DPT and YAP in cytoplasm of BT549 and MDA-MB-231 cells. *p < 0.05, **p < 0.01, ***p < 0.001, ****P < 0.0001.
