# Supplementary figures and images for: DNMT3a-dermatopontin axis suppresses breast cancer malignancy via inactivating YAP
Source: Cell Death Dis. 2023 Feb 11;14(2):106. doi: 10.1038/s41419-023-05657-8 (PMC9922281; doi:10.1038/s41419-023-05657-8)

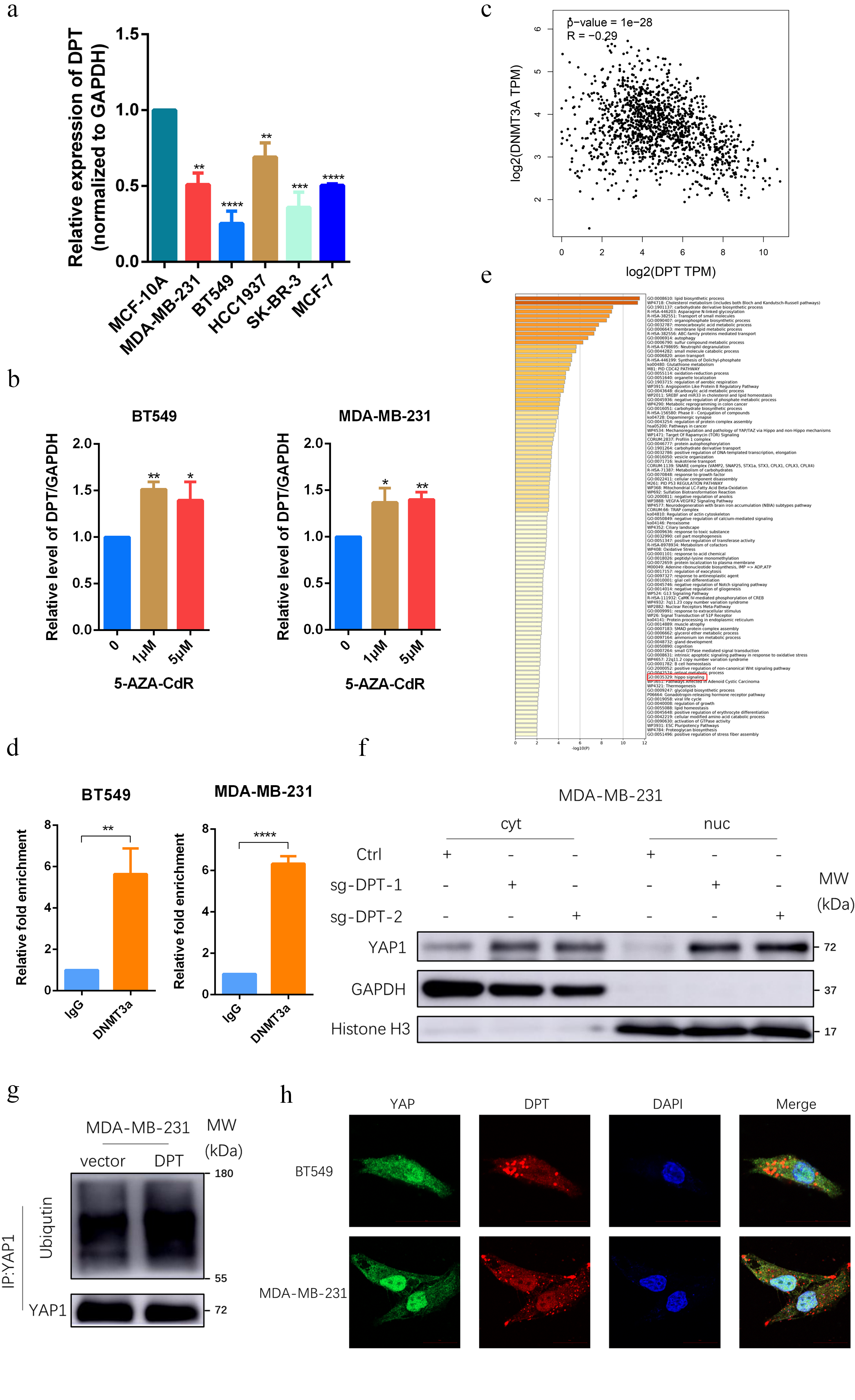

Supplement: Supplementary file 2 — Supplementary Figure 1 [file 41419_2023_5657_MOESM2_ESM.tif]
